# Video capsule endoscopy in patients with iron deficiency anaemia: experience at a regional Australian service

**DOI:** 10.1186/s13104-022-06053-9

**Published:** 2022-05-10

**Authors:** Maddison Furner, Robyn Nagel, Janani Pinidiyapathirage

**Affiliations:** 1 Logan Hospital, Meadowbrook, QLD 4131 Australia; 2 School of Medicine and Dentistry, Griffith University, Gold Coast, QLD 4222 Australia; 3 Rural Medical Education Australia, Toowoomba, QLD 4222 Australia

**Keywords:** Video capsule endoscopy, Iron deficiency anaemia, Regional Australia

## Abstract

**Objective:**

The objective of this study was to identify the diagnostic performance of video capsule endoscopy (VCE) among patients presenting with iron deficiency anaemia (IDA) and negative bidirectional endoscopy to a gasteroendoscopy practice in regional Australia. The secondary objectives were to identify the distribution of findings and factors predictive of positive findings in a regional setting.

**Results:**

In total 123 procedures were included in the study. Mean age of the patients was 67.9 years. Females made up 60.2% (n = 74) of the study population. Mean haemoglobin and ferritin levels were 93.3 g/L and 11.9 ug/L, respectively. Positive findings were present in 67 procedures (54.5%) with the most frequent finding being small bowel angiodysplasia (53.7%, n = 36/67), followed by ulceration/significant erosion (26.8%, n = 18/67), fresh blood (20.8%, n = 14/67) and tumour/polyp (16.4%, n = 11/67). Haemoglobin level was the only variable associated with positive findings (p = 0.005) in the study population. Of the procedures reporting positive findings outside the small bowel, the majority (80%) were within reach of conventional upper endoscopy and may have implications for future practice, particularly when allocating health resources in a rural setting.

## Introduction

Suspected small bowel bleeding (SSBB) accounts for 5–10% of all patients presenting with gastrointestinal bleeding and is suspected in patients with persistent iron deficiency anaemia (IDA) despite normal upper and lower endoscopy [1]. Occult SSBB presents as IDA, whilst overt SSBB presents as visible bleeding (haematemesis, melena or hematochezia) with or without IDA [1]. Video capsule endoscopy (VCE) has been the procedure of choice to investigate SSBB since the early 2000s. However, few studies have examined the diagnostic yield of VCE and predictive factors in patients with IDA, and none within a regional Australian context [2] where accessibility to health services may be limited. Considerable variability in diagnostic yield exists [2, 3] and there is limited data on predictive factors of positive VCE findings in patients with IDA [3]. Associations between increased yield and greater age, severity of anaemia, anti-coagulants, non-steroidal anti-inflammatory drugs (NSAIDs) and various comorbidities have been suggested, however these findings need to be confirmed by further studies [4–8].

This study was performed at a regional gasteroendoscopy clinic in Australia. The primary objective was to determine the diagnostic yield of VCE in IDA in this setting where referral patterns differ from major urban centres. Secondary objectives were to characterise positive findings for comparison to existing literature, and to identify factors predictive of positive findings to aid selection of patients who would most benefit from VCE. Appropriate patient selection for VCE has particular importance in a regional setting given the impact which rurality has on access to specialist healthcare. The vast distances between Australian rural towns and major cities and the concentration of specialists in urban centres constitute major barriers to access for regional patients [9, 10]. Given that VCE is neither widely available nor readily accessible in a regional setting, ideal selection of patients is imperative.

## Main text

### Methods

We conducted a retrospective review of electronic medical records of all VCE procedures performed at a private gastroenterology practice in regional Australia from March 2017 to April 2020. All procedures were performed using the Pillcam SB3 and analysed using the Medtronic rapid reader software by an experienced gastroenterologist.

To be included in the study patients had to meet the following inclusion criteria: age over 18 years, laboratory proven IDA (haemoglobin < 120 g/L for women, < 130 g/L for men and ferritin < 30 ng/L) [11], and a negative bidirectional endoscopy in the 12-months prior to VCE. Severity of anaemia was classified according to haemoglobin levels: mild (upto 110 g/L), moderate (< 110–80 g/L) and severe (< 80 g/L) using cut-offs defined by the World Health Organisation [12]. Exclusion criteria were: any pre-existing gastrointestinal conditions believed to be the cause of IDA, any extra-intestinal conditions believed to be the cause of IDA, known contraindication to VCE.

#### VCE procedure

Patients were instructed to cease oral iron medications three days prior to VCE and to have only a light diet the day prior to the procedure. On the day of the procedure, patients had to fast for 6-h, consume two litres of water 3-h before capsule ingestion and consume nothing for 3-h following the ingestion. Bowel preparation solutions were not routinely used.

#### Data collection

The patient’s demographic data and procedure reports were extracted to a secure database that included the indication for the procedure, gastric and small bowel transit times, findings and conclusions. Bowel visualisation was characterised as good or poor depending on whether the mucosa was visible for evaluation. Procedures with poor views were included only when significant findings were reported. A complete procedure was defined as the capsule passing through the ileocecal valve during its recording time of 12-h. Capsule retention was defined as non-passage of the capsule into the cecum within 2-weeks of ingestion. Eligible procedures were separated according to the Saurin criteria into positive and negative VCE findings [13]. Lesions categorised as P2 were considered a definite cause for IDA and were included in the positive results. P2 lesions included significant angioectasia, ulceration and widespread erosion, tumours, polyps or varices, fresh or active bleeding and significant findings outside the small bowel. Negative procedures included P1 lesions of uncertain bleeding potential and P0 findings indicated normal procedures.

### Statistical analysis

Normality tests were initially carried out to study the distribution of variables. Continuous data was presented as mean (95% confidence interval using the t-distribution) or median (interquartile range) for normally distributed and skewed data, respectively. An unpaired student t-test (for normally distributed data) or the Wilcoxon rank sum test (for skewed data) was used for comparing the distribution of variables among those with positive and negative endoscopy findings. Proportional differences were assessed using a Pearson’s χ^2^ test or Fisher’s exact test. A *p*-value < 0.05 was considered as statistically significant. IBM SPSS Statistical Software Program (Version 25) was used for the analysis.

### Results

Of the 297 procedures performed during the study period, 123 procedures performed in 120 patients met the study inclusion criteria. Three were repeated procedures for suspected missed lesions; six reported poor views. Due to incomplete data, 156 studies were ineligible for inclusion. Two patients were excluded as they were aged < 18 years, another 15 patients were excluded as they underwent VCE for indications other than IDA or were previously diagnosed as having coeliac disease (n = 3).

#### Baseline characteristics

The mean age of the study population was 67.9 years. There was a greater representation of females than males (60.2% versus 39.8%, p = 0.162, Table 1). The mean haemoglobin was 93.3 g/L (range 40 g/L to 129 g/L, Table 1). There were 36 (29.3%) patients with severe anaemia, 52 (42.3%) with moderate anaemia and 35 (28.4%) with mild anaemia (Table 2). The mean ferritin was 11.9 ug/L (range 2–30 ug/L, Table 1).

**Table 1:** Comparison of the clinical profile and examination findings among the study population

Variables	*Total study population (n* = *123)*	*Positive findings (P2) (n* = *67)*	*No positive findings (P1, P0) (n* = *39)*	*p-value*
Mean age	67.92	69.10	66.50	0.162
Male: female	49:74	30:37	19:37	0.221
Mean haemoglobin (g/L)	93.33	88.42	99.21	**0.005**
Mean ferritin (ug/L)	11.93	11.93	11.92	0.992
Mean Gastric Transit time (minutes)	54.53	40.37	73.21	0.137
Mean Small Bowel Transit time (minutes)	279.0	292.27	264.41	0.195
Completion rate (caecum visualised)	92.7%	93.7%	91.1%	0.730

Significant findings are given in bold

**Table 2:** Association of demographic and clinical factors with video capsule endoscopy findings

Patient characteristics	Total *n* (%)	With positive findings		Without positive findings	
		*n*	% (95% CI)	*n*	% (95% CI)
Total	123	67	54.5 (45.3–63.5)	56	45.5 (36.5–54.8)
Age					
< 60 years	23 (18.7)	8	34.8 (16.4–57.3)	15	65.2 (42.7–83.6)
≥ 60 years	100 (81.3)	59	59.0 (48.7–68.7)	41	41.0 (31.3–51.3)
Type of bleeding					
Occult	85 (69.1)	48	56.5 (45.3–67.2)	37	43.5 (32.8–54.7)
Overt (melena)	23 (18.7)	13	56.5 (34.5–76.8)	10	43.4 (23.2–65.5)
Overt (bright)	6 (4.9)	2	33.3 (4.3–77.7)	4	66.7 (22.3–95.7)
Haemoglobin (g/L)					
Mild	35 (28.5)	16	45.7 (28.8–63.4)	19	54.3 (36.7–71.2)
Moderate	52 (42.3)	24	46.2 (32.2–60.5)	28	53.8 (39.5–67.8)
Severe	36 (29.3)	27	75 (57.8–87.9)	9	25.0 (12.1–42.2)
Ferritin (ug/L)					
≥ 15	30 (24.4)	13	43.3 (25.5–62.6)	17	56.7 (37.4–74.5)
< 15	64 (52.0)	32	50 (37.2–62.8)	32	50 (37.2–62.8)
NSAID: yes	62 (50.4)	32	51.6 (38.6–64.5)	30	48.4 (35.5–61.4)
Transfusion: yes	19 (15.4)	11	57.9 (33.5–79.8)	8	42.1 (20.3–66.5)
Iron replacement: yes	57 (46.3)	27	47.4 (33.9–61.0)	30	52.6 (39.0–66.0)
Diabetes: yes	27 (22.0)	15	55.6 (35.3–74.5)	12	44.4 (25.5–64.7)

Abbreviations: NSAID: non-steroidal anti-inflammatory drugs; CI: Confidence Interval

#### Positive VCE findings

The diagnostic yield of VCE in this study was 54.5% (n = 67/123, Table 1). There were 67 procedures with a total of 116 positive findings considered clinically significant and the likely cause of IDA. The most common finding within the small bowel was angiodysplasia (n = 36). Other significant findings within the small bowel included ulceration/significant erosion (n = 18), tumour/polyp (n = 11) and fresh blood (n = 14). One study demonstrated lymphangioectasia and one reported portal enteropathy. A number of procedures had multiple significant findings (n = 34, 27.6%) with 28 reporting 35 significant findings outside of the small bowel. Of these, 16 reported significant small bowel lesions in addition to lesions in other parts of the gut. Of the 35 lesions detected outside the small bowel, 28 (80.0%) were detected within reach of standard upper endoscopy. Due to the clinical relevance of significant findings outside the small bowel, all findings were included in the calculation of diagnostic yield of VCE.

#### Factors predictive of positive VCE findings

A range of demographic and clinical factors were analysed to assess for predictive factors of positive findings on VCE. Mean haemoglobin was significantly associated with positive findings (*p* = 0.005, Table 1). However, ferritin which is another marker of IDA severity or any of the other selected factors did not show a significant association with positive findings.

## Discussion

The overall diagnostic yield of this study was 54.4%. The diagnostic yield of VCE in IDA is highly variable; a recent systematic review reported a pooled diagnostic yield of 66% (range 32.5–77.7%) [2]. Variability in yield likely relates to the lack of uniform criteria for positive findings amongst other factors. To mitigate this, in this study a single gastroenterologist reported all procedures and the Saurin criteria were utilised to define positive findings. Appropriate patient selection and a high caecal visualisation rate (92.5%) may have contributed to the high yield reported in this study.

The most common finding within the small bowel in this study was angiodysplasia (n = 36/67, 53.7%). Angiodysplasia has widely been reported as the most common finding on VCE in Western settings [2, 3, 14]. In a systematic review of VCE in IDA a detection rate of 45.9% for angiodysplasia was reported, this is comparable to the rate in this study [2]. Ulceration was the next most common finding, consistent with the rate of NSAID use in the study population (n = 62, 50.4%, Table 2). Of importance was the frequency of significant findings outside of the small bowel. Yung et al. found 42% of positive studies had significant lesions within reach of standard endoscopy [7]. Other studies have reported lower rates ranging 3.5–30% [15]. Differences between study criteria determining positive findings outside the small bowel likely accounts for some of this variability, however the high rate of “missed lesions” highlights the importance of the initial endoscopic assessment. This is particularly relevant for rural patients who encounter increased logistical and financial barriers to pursuing specialist services such as VCE [10]. The results of this study may support the claim for second look endoscopy prior to VCE suggested by others [16, 17]. Interestingly, contrary to the results of other studies, the majority of lesions detected outside the small intestine in this study were within range of upper endoscopy, rather than in the large bowel [15].

This study found a statistically significant association between mean haemoglobin and positive findings on VCE (Table 1). Previous studies have identified haemoglobin level as having an independent effect on the probability of obtaining positive findings [8]. Low haemoglobin is thought to indicate greater or more persistent blood loss over time and thus lesions detectable on VCE. Older age and male gender have also been associated with increased diagnostic yield [4, 18]. These factors were not significantly associated with diagnostic yield in our study, however we found a trend for positive findings in both these groups. Concomitant anticoagulant/antiplatelet therapy, NSAID use, increased small-bowel transit time, history of overt bleeding and various comorbidities have also been associated with diagnostic yield, however, were not found to predict yield in this study [5, 19, 20]. Future studies should focus on these parameters to further identify patient’s most likely to benefit from VCE.

## Conclusion

Our study suggests that the diagnostic yield of patients with IDA in a regional Australian setting is comparable to internationally reported data. Patients with severe anaemia, as reflected by haemoglobin level, were more likely to have positive findings on VCE and should be referred as a priority. The high proportion of findings within reach of conventional endoscopy also emphasises the importance of the initial endoscopic assessment and second-look endoscopy prior to VCE in resource limited settings.

## Limitations

The main limitation of our study was its retrospective design and consequent incomplete data and modest sample size. To be included in this study, laboratory evidence of iron deficiency anaemia in the twelve months prior to VCE was required, this contributed to exclusion of some cases due to incomplete data. Reasons for incomplete data were multifactorial, but primarily reflected local referral patterns. Referrals were received from private specialists, local public hospital and rural and remote general practitioners and did not always contain the relevant laboratory data. Due to resource limitations, requesting additional data from the original referrers was not possible. In addition, information on further management was not always available, hence analysis of outcomes was not attempted in this study. Another limitation was that several proposed predictive factors could not be examined due to incomplete data.

The experience at the practice was that adequate views could be obtained in most patients with dietary modification alone, hence routine bowel preparation was not done. Recent consensus guidelines indicate that oral bowel preparation has benefits in terms of visualisation that were “sufficient to recommend its use”, though there is insufficient evidence to recommend a particular type of preparation [21]. The absence of bowel preparation in this study may have affected diagnostic yield, however the low rate of studies with poor views (6/123 studies, 4.8%) suggests that any potential impact was minimal.

The European Society of Gastrointestinal Endoscopy identifies a target of 90% of patients undergoing VCE within 14 days of an overt bleeding event as a key performance indicator [14]. In this study, patients were often referred from rural areas following an initial work-up locally, and delays to VCE following negative bidirectional endoscopy were not uncommon. Despite this, the study reported a high diagnostic yield.

## Data Availability

The de-identified dataset used in the current study could be made available on reasonable request from the Toowoomba Gastroenterology Clinic, robynnagel@tgclinic.com.au.

## Abbreviations

ESGE: European Society of Gastrointestinal Endoscopy
GAVE: Gastric antral vascular ectasia
IDA: Iron deficiency anaemia
NSAID: Non-steroidal anti-inflammatory drugs
SSBB: Suspected small bowel bleeding
VCE: Video capsule endoscopy
